# Measurement Properties of Caregiver Impact Measures in Pediatric Chronic Gastrointestinal Disorders: A Systematic Review

**DOI:** 10.21203/rs.3.rs-10606914/v1

**Published:** 2026-08-25

**Authors:** Daniela Gattini, Camille Maltais-Bilodeau, Cornelia M. Borkhoff, Jessie Cunningham, Yaron Avitzur, Wendy J. Ungar, Paul W. Wales

**Affiliations:** Hospital for Sick Children, University of Toronto; Centre Hospitalier Universitaire de Sherbrooke (CHUS), Université de Sherbrooke; Hospital for Sick Children, University of Toronto; Hospital for Sick Children; Hospital for Sick Children, University of Toronto; Hospital for Sick Children, University of Toronto; University of Cincinnati

**Keywords:** Caregiver burden, pediatric gastrointestinal disorders, patient-reported outcome measures, psychometric properties, measurement properties, systematic review

## Abstract

**Purpose:**

Caregivers of children with chronic gastrointestinal disorders experience physical, psychological, social, and financial impact. This systematic review aimed to evaluate the available evidence on the measurement properties of caregiver impact PROMs in chronic gastrointestinal disorders and to identify the best available measures.

**Methods:**

MEDLINE, Embase, CINAHL, PsycINFO, and Scopus were comprehensively searched from their inception to April 30, 2026. All English language studies examining the measurement properties of caregiver impact measures in children with chronic gastrointestinal diseases were included, irrespective of measure language. All COSMIN measurement properties were examined. Risk of bias was assessed to derive recommendations for suitable PROMs to measure caregiver impact.

**Results:**

A total of 24 studies of 26 tools were included. Study quality was moderate or low. Measures with sufficient evidence of validity included: Arabic Celiac Disease Parent Caregiver Quality of Life (CDPCA-QoL); Celiac Disease Life Inventory of Family Experiences (CDLIFE); CDPCA-QoL; Cincinnati Fecal Incontinence Scale (CINCY-FIS); Croatian Pediatric Quality of Life Inventory^™^ Family Impact Module (PedsQL^™^ FIM); Feeding/Swallowing Impact Survey (FS-IS); Portuguese FS-IS; Parental Opinions of Pediatric Constipation (POOPC); Work Productivity and Activity Impairment (WPAI)-Crohn’s Disease-caregiver; and WPAI-Ulcerative Colitis-caregiver. The review identified 16 additional PROMs assessing caregiver impact; however, further research is required to determine the adequacy of their measurement properties.

**Conclusion:**

Despite the number of caregiver impact PROMs identified, high-level evidence supporting their measurement properties in chronic gastrointestinal disorders remains limited. Ongoing measurement evaluation and rigorous cultural validation are essential to strengthen the evidence base and support their broader clinical application.

## Plain English Summary

Parents and other caregivers of children with chronic gastrointestinal conditions often experience emotional, physical, social, and financial challenges. Understanding these experiences is important for providing appropriate support and for evaluating whether treatments improve not only children’s health but also family well-being. To do this, researchers and healthcare providers need questionnaires that accurately measure the impact of caregiving. However, it has been unclear which questionnaires are reliable and suitable for use.

This systematic review examined all available studies evaluating questionnaires that measure caregiver impact in pediatric chronic gastrointestinal disorders. We identified 26 questionnaires reported in 24 studies and assessed how well they measured caregiver experiences using internationally recognized standards. Although 10 questionnaires demonstrated good evidence of validity, the overall quality of the evidence was generally moderate or low, and many measures require further evaluation before they can be recommended for widespread use.

Our findings show that more rigorous validation studies are needed to strengthen these questionnaires. Better measurement tools will improve research, support clinical care, and help ensure that the impact of childhood gastrointestinal diseases on families is recognized and addressed.

## Introduction

Caregivers are defined as persons that provide essential support and medical care to people with cancer, disability, injury, or chronic conditions in home and community-based settings.[1] Chronic debilitating pediatric conditions, such as gastrointestinal disorders, have significant spillover effects on caregivers.[2–5] Caregiver impact is a broad construct encompassing both the potentially positive experiences and the negative impacts (sometimes referred to as “burden”) related to caregiver duties. Caregiver “burden” has been defined by Zarit et al. as “the extent to which caregivers perceive that caregiving has had an adverse effect on their emotional, social, financial, physical and spiritual functioning.”[6] This multidimensional concept incorporates emotional, physical, social, and economic aspects of the caregiving role, considered to be unique for each caregiver.[7]

Evaluation of caregiver impact represents an important area of research in pediatric gastroenterology. Long-term caregiving for a child or adolescent with a chronic gastrointestinal disease may negatively impact physical and mental health.[8, 9] Caregivers of children with chronic gastrointestinal diseases are at an increased risk of financial challenges, and psychological consequences such as anxiety, stress, and depression.[1, 4, 5] Furthermore, caregiver quality of life is increasingly recognized as a critical outcome in the treatment of children with chronic gastrointestinal disorders.[10–12]

Multiple patient-reported outcome measures (PROMs) have been developed to measure caregiver impact and include different domains of the construct, such as the PedsQL Family Impact Module (FIM), the Impact on Family Scale (IFS), Pediatric Inventory for Parents (PIP), and the Family Management Measure (FaMM).[13–18] However, it is important to determine the validity, reliability, and responsiveness of these PROMs for use in caregivers of children with chronic gastrointestinal diseases and assess if these tools can be adequately applied in this patient population. This review aimed to systematically review the literature and derive recommendations for rigorously developed and validated PROMs assessing caregiver impact in pediatric chronic gastrointestinal diseases.

## Methods

This systematic literature review was conducted following the Preferred Reporting Items for Systematic Reviews and Meta-analyses (PRISMA) reporting guidelines (**Supplementary Material 1**),[19] as well as the COnsensus-based Standards for the selection of health Measurement Instruments (COSMIN), PRISMA-COSMIN reporting guidelines developed specifically for systematic reviews of PROMs.[20] The review was not registered in PROSPERO since it was a Methodology Review (not included in PROSPERO currently). Ethics approval was not required, since this systematic review relies exclusively on previously published literature and publicly available data.

### Inclusion and exclusion criteria for the review

All published peer-reviewed English language studies examining the measurement properties of caregiver impact measures in caregivers of children and adolescents with chronic gastrointestinal diseases were considered for inclusion, irrespective of the language in which the instruments were administered. The review included validation studies with evidence of PROM development or any other study design assessing any of the following nine measurement properties defined in the COSMIN guidelines: content validity, structural validity, internal consistency, cross-cultural validity, reliability, measurement error, criterion validity, hypothesis testing for construct validity, and responsiveness.[21–24] Table 1 provides operational definitions of these terms. According to the COSMIN methodology, content validity is the most important measurement property. Once established, the other properties should be evaluated to determine the overall quality of an instrument and prioritized based on the intended use of the tool.[24]

Studies were excluded if they did not assess caregiver impact; if they measured caregiver impact in caregivers of adult patients (above 18 years of age); if they assessed caregiver impact in other patient populations; if they did not specify whether children with chronic gastrointestinal disorders were included; and if they did not report measurement properties of the included PROM. Descriptive articles, abstracts with no full-text available, and studies published in languages other than English were excluded.

### Information sources and search strategies

The search strategy was developed by a professional medical librarian (JC) trained in knowledge synthesis. The search was executed in the following bibliographic databases from inception to March 27, 2024, and then updated in May 2026 to retrieve publications from end of the previous search to April 30, 2026: Ovid MEDLINE(R) and Epub Ahead of Print, In-Process, In-Data-Review & Other Non-Indexed Citations and Daily; Ovid Embase+Embase Classic; Ovid APA PsycInfo; Ebsco CINAHL Plus with Full Text; and Elsevier SCOPUS. The search strategy consisted of both controlled vocabulary, such as the National Library of Medicine’s MeSH (Medical Subject Headings) and Emtree Subject Headings (Embase), PsycINFO Subject Headings, CINAHL Subject headings, and keywords. Results were limited to English language publications. See **Supplementary Material 2** for the detailed search strategy.

### Data collection process

Article abstracts and full text were imported into Covidence systematic review software (Veritas Health Innovation, Melbourne). Two reviewers (D.G. and C.M.B.) screened all identified titles and abstracts for potential eligibility independently. The two reviewers retrieved and reviewed full texts that included evaluations of caregiver impact tools in gastrointestinal disorders and assessed them for inclusion independently. Any disagreements were resolved by discussion with a third reviewer (C.B.). Reference lists from all identified manuscripts were examined manually for additional potentially eligible studies. A systematic approach to data extraction was used to produce a descriptive summary of the measurement properties for each outcome measure. Data extraction focused on the measurement properties included in the COSMIN checklist.[21]

Caregiver impact was the primary outcome measure. Types of caregiver impact instruments included were caregiver reported measures (PROMs) of health-related quality of life (HRQOL), physical effects, psychological effects, social effects, and financial impacts. Characteristics of the included studies and PROMs included: specific caregiver population, country, PROM language, type of study, study sample size, domains, items, recall period, Likert-scale, score range, and instrument availability. Chronic gastrointestinal disorders were defined as long-standing conditions of the digestive system, with symptoms persisting for ≥ 3 months, that may involve functional, inflammatory, structural, or motility-related abnormalities and are associated with sustained health effects.

### Risk of bias and measurement properties in the included studies

The COSMIN risk of bias checklist[21] was used to assess the methodological quality of the included studies and PROMs. The COSMIN checklist rates the rigour of each psychometric domain, required for the adequate use of health-related PROMs.[21–23] Each psychometric domain consists of two to eight items rated on a five-point Likert scale from 0 to 4, i.e. “not available,” “inadequate,” “doubtful,” “adequate,” or “very good.” A worst score counts principle was applied for each domain, so that the overall evidence rating was equal to the lowest score in the Likert-scale for that domain (e.g. if one item was rated as “inadequate,” the entire domain was rated as “inadequate” even if other items were “adequate”).[21] Only those domains which were evaluated in a particular study were assessed.

As suggested by COSMIN guidelines, the overall performance ratings from all studies evaluating each of the measurement properties were then rated as either sufficient (+), insufficient (−), or indeterminate (?) if not enough information for that measurement property was available.[21] If more than one study assessed a psychometric measurement property for the same instrument, individual ratings from the assessment of measurement properties from individual studies were pooled to provide an overall performance rating of a PROM for that measurement property.

### Best evidence synthesis

A level of evidence was assigned for the psychometric measurement properties of each PROM using a modified Grading of Recommendations Assessment, Development, and Evaluation (GRADE) approach for systematic reviews of clinical trials.[25] The level of evidence was rated as high, moderate, low, or very low depending on the following factors: risk of bias; inconsistency; imprecision; and indirectness, according to COSMIN guidelines.[21, 23, 25] If only one study was available for a particular PROM, the GRADE assessment was downgraded. Finally, a summary of the level of evidence for each PROM was presented, with a level of recommendation of class A (recommended), B (further research required), or C (not recommended).

A systematic approach was used by two independent reviewers (D.G and C.M.B.) to rate the quality or overall level of evidence supporting each measure and risk of bias. Any disagreements were resolved by discussion including the third reviewer (C.B.). Results were reported narratively given the wide differences in constructs and patient populations between studies.

## Results

**Figure 1.**
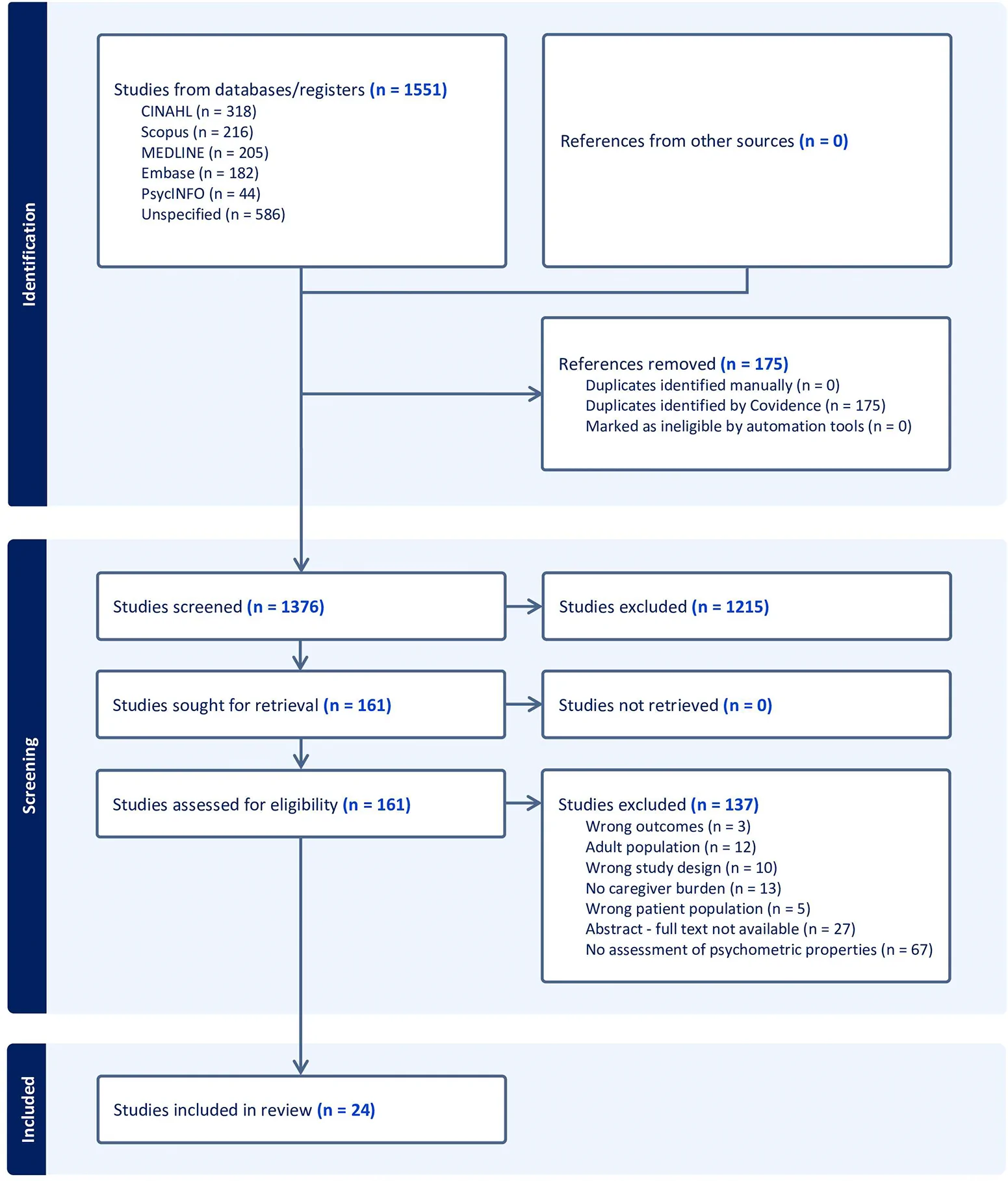
PRISMA flow diagram of the study selection process.

The systematic literature search yielded a total of 1551 publications; 175 were excluded as duplicates. Of the remaining 1376 publications, 1215 were eliminated due to not being relevant for the review. Full-text reviews of the 161 remaining studies resulted in 24 eligible studies. No further studies were identified after checking the listed references of the included studies. The most common reasons for ineligibility were studies not assessing psychometric properties of PROMs used and abstracts that did not have a full-text available. The PRISMA flowchart (Fig. 1) illustrates the study selection process and lists the reasons for exclusion after full-text analysis.

### Study characteristics

The characteristics of the included studies are summarized narratively in Table 2. The 24 studies revealed psychometric property evidence for a total of 26 tools assessing caregiver impact. The included PROMs assessed caregiver impact in studies of caregivers of children or adolescents with celiac disease, inflammatory bowel disease (IBD), irritable bowel syndrome (IBS), functional abdominal pain disorders, severe constipation and fecal incontinence, gastroesophageal reflux disease (GERD), food protein induced enterocolitis (FPIES), feeding or swallowing problems, and short bowel syndrome (SBS).

The systematic review found seven cross-sectional studies that assessed the development and validation of instruments that measured caregiver impact: the Celiac Disease Parent Caregiver Quality of Life (CDPCA-QoL),[26] the Celiac Disease Life Inventory of Family Experiences (CDLIFE);[27] the Cincinnati Fecal Incontinence Scale (CINCY-FIS),[28] the Feeding/Swallowing Impact Survey (FS-IS),[29] Parental Opinions of Pediatric Constipation (POOPC), the Patient-Reported Experience and Outcome Measure in a Bowel Management Program (PREOM-BMP);[30] and the Fecal Incontinence and Constipation Quality of Life (FIC QOL).[31] The PREOM-BMP study assessed the patient and caregiver’s experience and outcomes in bowel management programs for severe constipation and fecal incontinence.[30] The FIC QOL is a fecal incontinence and constipation quality of life measure for children with spina bifida and their caregivers.[31] Four studies validated previously developed instruments in different cultures and languages: the Croatian version of the Pediatric Quality of Life Inventory^™^ Family Impact Module (PedsQL^™^ FIM) in caregivers of children with IBD and celiac disease,[32] the Portuguese version of the FS-IS in caregivers of children with feeding and swallowing problems,[33] the Arabic version of the CDPCA-QoL,[34] and the Spanish version of the Work Productivity and Activity Impairment-Crohn’s Disease (WPAI-CD)-caregiver and the Work Productivity and Activity Impairment-Ulcerative Colitis (WPAI-UC)-caregiver.[35] Furthermore, the review found two qualitative studies on the initial development of PROMs assessing caregiver burden providing evidence of content validity that require further validation studies to examine their psychometric properties and to refine the items and develop an appropriate scoring model. These studies included the simultaneous development of the American English and American Spanish Pediatric GERD Caregiver Impact Questionnaire (PGCIQ),[36] and the development of a pilot instrument combining the previously validated generic Family Management Measure (FaMM) with a SBS-specific quality of life questionnaire.[13]

Additionally, 11 studies applied 12 PROMs, previously validated in other caregiver populations, to caregivers of children with chronic gastrointestinal disorders, IBD, celiac disease, functional gastrointestinal disorders, and FPIES. These studies provided evidence of internal consistency: the Pediatric Inventory for Parents (PIP);[37–41] the Stigma Scale Parent (SS-P);[42] the Illness Intrusiveness Scale Parent (IIS-P);[42] the RAND-36 Item Health Survey 1.0 (RAND-36);[43] the Generalized Anxiety Disorder-7 (GAD-7);[44] the 10-Item Perceived Stress Scale (PSS-10);[44] the Health Anxiety by Proxy Scale (HAPYs); [45] the Symptom Checklist-8 (SCL-8);[45] the Food Allergy Quality of Life–Parental Burden (FAQL-PB);[46] the Arabic version of the Multidimensional Scale of Perceived Social Support (MSPSS);[47] the Arabic version of the PSS-10;[47] and the Arabic version of the Zarit Burden Interview (ZBI-A).[47]

### PROM Characteristics

Characteristics of each included instrument are summarized in Table 3. The PROMs identified assessed different constructs of caregiver impact including physical, emotional, and social well-being, worry, family functioning, relationships, daily activities, caregiver management ability and effort, difficulties with the medical team, financial impact, and employment. **Supplementary Material 3** summarizes the different constructs comprised in the included PROMs.

Number of domains and items varied across different instruments. Most PROMs used a 5-point Likert rating scale to determine a score range. Only 11 PROMs reported their recall periods. Eleven PROMs are freely available to use for research purposes. The other PROMs have a cost associated with its use, or the tool is not readily available to other researchers.

### Measurement properties

Table 4 summarizes the psychometric measurement property ratings for each PROM based on overall ratings of pooled results from all studies. Internal consistency was evaluated in 23 (88.5%) of the included PROMs, while only 10 (38.5%) had at least moderate level of evidence for both structural validity and internal consistency (Table 4). Regarding internal consistency for all instruments, Cronbach’s α values ranged from 0.658 (WPAI-UC-Caregiver)[35] to 0.96 (POOPC).[48] The accepted threshold of at least 0.70 for a scale is considered sufficiently reliable.[49] Only four studies had cross-cultural validity evidence, [32–35] and only four had evidence for test-retest reliability.[26, 28, 31, 48] Only one of the PROMs assessed responsiveness.[26]

Table 4 provides a GRADE level of evidence for the overall rating of each measurement property for each PROM. The level of evidence was moderate, low, or not available for most of the measurement properties of the PROMs assessed. Studies were frequently downgraded for insufficient number of studies validating the PROM (i.e., only one study) or low numbers of study patients (< 100), resulting in imprecision. **Supplementary Material 4** provides justification for downgrading where applicable.

Of the 26 PROMs identified, the following 10 measures received a class A recommendation level based on adequate content validity, a low/moderate level of evidence for internal consistency, and sufficient structural validity: Arabic version CDPCA-QoL; CDLIFE; CDPCA-QoL; CINCY-FIS, Croatian version PedsQL^™^ FIM, FS-IS, Portuguese version FS-IS, POOPC, WPAI-CD-caregiver; and WPAI-UD-caregiver. Of these, the CDLIFE, CINCY-FIS, Croatian version PedsQL^™^ FIM, WPAI-CD-caregiver and WPAI-CD-caregiver are freely available for use. The remaining 16 PROMs demonstrated sufficient content validity but did not demonstrate sufficient internal consistency or had sufficient internal consistency but insufficient evidence of content validity and therefore received a class B recommendation. None of the PROMs received a class C recommendation.

## Discussion

This is the first systematic review that used COSMIN guidelines to assess measurement properties for caregiver impact PROMs in pediatric chronic gastrointestinal disorders. The review found that the Arabic version CDPCA-QoL; CDLIFE; CDPCA-QoL; CINCY-FIS, Croatian version PedsQL^™^ FIM; FS-IS; Portuguese version FS-IS; POOPC; WPAI-CD-caregiver; and WPAI-UD-caregiver have sufficient evidence of measurement properties and can be recommended for clinical use. Most of the recommended PROMs were developed and validated for specific populations with unique needs including celiac disease, constipation, fecal incontinence, and swallowing or feeding difficulties, and may not be relevant for other chronic gastrointestinal disorders.[26–29, 33–35, 48] On the other hand, the Croatian version of the PedsQL FIM that was validated on patients with inflammatory bowel disease and celiac disease, could also be used in other similar gastrointestinal disorders.[50] The review identified an additional 16 PROMs that lack sufficient evidence of measurement properties.

There is growing literature highlighting caregiver impact in caregivers of patients with chronic gastrointestinal disorders and different tools have been utilized to measure caregiver impact in this patient population.[9, 43, 51–53] A study of 102 caregivers of adults with IBD found that 39% of IBD caregivers experienced burden as assessed by the Zarit Burden Interview Score (ZBI).[51] Factors associated with negative impacts included caregiver white race, patient history of fistulas, diagnosis of ulcerative colitis, higher caregiver education, and more hours spent caregiving.[51] Greater disease activity is associated with lower parent quality of life in mental health and physical health domains.[43] In a study by Engstrom et al, mothers of children with IBD scored very high on parental distress using the Symptom Check-List 90 (SCL-90) instrument, whereas the fathers did not differ from the comparison group. [9] This study did not include assessment of measurement properties.[9] Several studies have also reported increased parental stress and caregiver burden in caregivers of patients with dysphagia, GERD, eosinophilic gastrointestinal disorders, gastroparesis, celiac disease, chronic constipation, fecal incontinence, intestinal failure, and cystic fibrosis measured with the instruments included in this review as well as the PedsQL FIM and the FaMM.[4, 26, 29–31, 48, 54–60] However, most studies did not provide evidence of validity or other measurement properties.

The type of caregiving relationship can influence caregiver impact. A study comparing adult-child and spousal caregiver burden using the ZBI score reported that adult-child caregivers experienced higher social/emotional burden and financial burden, compared with spouses.[61] Risk factors for caregiver burden included residing with the care recipient, higher number of hours spent caregiving, and financial stress.[62] Amongst mothers of children with chronic conditions, child’s number of medicines and injections, a diagnosis of attention-deficit/hyperactivity disorder in addition to the primary medical condition, frequent visits with a primary care provider and emergency room visits, and lower child self-efficacy were associated with increased caregiver burden (ZBI score).[63] Furthermore, there has been increased recognition that patients with various chronic illnesses are impacted not only by their disease but also the treatments required to manage their condition.[30] Sometimes, the negative impacts of treatment can match that of the manifestation of the disease itself. [64] As children are largely reliant on caregivers, this experience often extends to family members, placing significant psychological, social, and financial strain on those involved.[65] Patients and parents who experience a high burden of therapy may struggle to adhere to prescribed treatments, which may trigger nonadherence, frequent hospitalizations, higher healthcare costs, and increased morbidity.[66, 67] Therefore, it is recommended that health care providers remain vigilant to the impact of caring for a child with chronic gastrointestinal disorders, and consider implementing interventions when necessary.

The included studies have limitations. First, most studies had limited measurement property evidence and included mainly evidence of content validity and/or internal consistency. Additionally, even the studies that had sufficient evidence of content validity, structural validity, and internal consistency, were rated as moderate or low-quality evidence as per GRADE recommendations. This was mainly due to limited number of studies assessing each PROM. Another limitation is the heterogenous populations included in different studies. Some gastrointestinal disorders have similarities (e. g., diarrhea, vomiting), while certain disorders have unique characteristics that can impact quality of life of patients and caregivers (e. g., stomas, feeding tubes, central lines, intravenous medication requirements). These particularities should be considered when selecting the most appropriate caregiver impact instrument. Limitations of the review included English language restriction and potential selection bias due to search strategy and publication bias. However, the review included studies of measures developed or validated in other languages that were written in English.

In conclusion, the Arabic CDPCA-QoL, CDLIFE, CDPCA-QoL, CINCY-FIS, Croatian PedsQL^™^ FIM, FS-IS, Portuguese FS-IS, POOPC, WPAI-CD-caregiver, and WPAI-UC-caregiver demonstrated sufficient measurement properties and can be recommended for clinical use. Awareness and appropriate selection of validated caregiver impact measures may assist clinicians in quantifying caregiver HRQOL and impacts, recognizing caregivers who may benefit from additional support, ultimately improving outcomes for children with chronic gastrointestinal disorders.

This review highlights the need for ongoing psychometric evaluation and rigorous cultural validation of existing caregiver burden tools to strengthen their evidence base and support their broader clinical application. There is also a need for the development of disease-specific caregiver impact instruments, with active caregiver involvement throughout the tool development process to ensure relevance, acceptability, and content validity.

## Supplementary Material

Supplementary Files

This is a list of supplementary files associated with this preprint. Click to download.

• SupplementaryMaterial1PRISMA2020Checklist.docx

• SupplementaryMaterial2SearchStrategy.docx

• SupplementaryMaterial3.docx

• SupplementaryMaterial4.docx

## Figures and Tables

**Table 1:** COSMIN definitions of Measurement Properties

Measurement Property	Definition (COSMIN)
***Validity***	
Content Validity	The degree to which the content of an instrument is an adequate reflection of the construct to be measured. Includes relevance, comprehensiveness, and comprehensibility.
Structural Validity	The degree to which the scores of an instrument are an adequate reflection of the dimensionality of the construct to be measured (e.g., through factor analysis).
Cross-cultural validity / Measurement invariance	The degree to which the performance of the items on a translated/adapted instrument are an adequate reflection of the original version and comparable across different groups (e.g., cultures, languages).
Criterion Validity	The degree to which the scores of an instrument are an adequate reflection of a “gold standard.” Rarely applicable when no true gold standard exists.
Hypothesis testing for construct validity	The degree to which the scores of an instrument are consistent with predefined hypotheses based on the assumption that the instrument validly measures the construct. This can include convergent validity, divergent validity, and known-groups validity.
**Reliability**	
Internal Consistency	The degree of interrelatedness among items within a scale. Often assessed using Cronbach’s alpha.
Reliability	The degree to which an instrument is free from measurement error, reflecting the consistency of scores in patients who are stable over time. Examples include the test–retest reliability, intra-rater reliability, and inter-rater reliability.
Measurement error	The systematic and random error of a score not attributed to true changes in the construct being measured. Examples include the standard error of measurement (SEM) or smallest detectable change (SDC).
**Responsiveness**	
Responsiveness	The ability of an instrument to detect change over time in the construct to be measured, when change has occurred.

COSMIN, COnsensus-based Standards for the selection of health Measurement Instruments

*Note.* Adapted from “The COSMIN study reached international consensus on taxonomy, terminology, and definitions of measurement properties for health-related patient-reported outcomes” by LB. Mokkink, 2010, *Journal of Clinical Epidemiology*, 63, p.737–745.

**Table 2:** Characteristics of Included Studies

Study	PROMs Included	Caregiver Population	Country	Type of Study	Sample Size
Abreu Paiva, et al. 2019.	CDPCA-QoL	Children with celiac disease	Brazil	Cross-sectional Development and Validation	150
Al-Dala’ien, et al. 2026.	Arabic version CDPCA-QoL	Children with celiac disease	Jordan	Cross-sectional Validation	198
Almulla, et al, 2024.	Arabic MSPSS Arabic PSS-10 ZBI-A	Includes children with chronic gastrointestinal disorders	Saudi Arabia	Cross-sectional	206
Baudino, et al. 2021.	SS-P IIS-P	Adolescents with IBD	United States	Cross-sectional	150
Coburn, et al. 2026.	CDLIFE	Children with celiac disease	United States	Cross-sectional Development and Validation	103
Cushing, et al. 2018.	CINCY-FIS	Children with fecal incontinence	United States	Cross-sectional Development and Validation	222
Guilfoyle, et al. 2012.	PIP	Adolescents with IBD	United States	Cross-sectional	62
Gray, et al. 2013.	PIP	Adolescents with IBD	United States	Cross-sectional	130
Gray, et al. 2015.	PIP	Adolescents with Crohn’s disease	United States	Cross-sectional	99
Greenhawt, et al. 2016.	FAQL-PB	Children with FPIES	United States	Cross-sectional	68
Greenley, et al. 2009.	RAND-36	Adolescents with IBD	United States	Cross-sectional	55
Halleran, et al. 2019.	PREOM-BMP	Children with severe constipation and incontinence undergoing BMP	United States	Cross-sectional Development and Validation	359
Kim, et al. 2005.	PGCIQ	Children with GERD	United States	Qualitative Development	27 (12 English; 15 Spanish)
Knez, et al. 2015.	Croatian version PedsQL^™^ FIM	Children with IBD and celiac disease	Croatia	Cross-sectional Validation	212
Lefton-Greif, et al. 2014.	FS-IS	Children with feeding/swallowing problems	United States	Cross-sectional Development and Validation	164
Nanigian, et al. 2008.	FIC QOL	Children with SB, fecal incontinence, and constipation	United States	Cross-sectional Development and Validation	144
Neumann, et al. 2021.	SBS specific FaMM	Children with short bowel syndrome	Unites States	Qualitative Development of pilot tool	17
Pacut, et al. 2025.	GAD-7 PSS-10	Children of chronic gastrointestinal diseases	Poland	Cross-sectional	562
Rama, et al. 2020.	Portuguese version FS-IS	Children with feeding/swallowing problems	Brazil	Cross-sectional Validation	95
Rea, et al. 2022.	PIP	Children with Crohn’s disease	United States	Cross-sectional	53
Robbertz, et al. 2022.	PIP	Children with IBD, celiac disease or IBS	United States	Cross-sectional	185
Skovslund Nielsen, et al. 2025.	HAPYs SCL-8	Youths with functional abdominal pain disorders	Denmark	Cross-sectional	87
Velasco Rodriguez-Belvis, et al. 2024.	WPAI-CD-Caregiver WPAI-UC-Caregiver	Children with IBD	Spain	Cross-sectional Validation	524
Silverman, et al. 2015.	POOPC	Children with constipation	United States	Cross-sectional Development and Validation	410

Abbreviations: BMP, bowel management program; CDLIFE, Celiac Disease Life Inventory of Family Experiences; CDPCA-QoL, Celiac Disease Parent Caregiver Quality of Life; CINCY-FIS, Cincinnati Fecal Incontinence Scale; FaMM, Family Management Measure; FAQL-PB, Food Allergy Quality of Life–Parental Burden; FIC QOL, Fecal Incontinence and Constipation Quality of Life; FPIES, food protein induced enterocolitis; FS-IS, Feeding/Swallowing Impact Survey; GAD-7, Generalized Anxiety Disorder-7; GERD, gastroesophageal reflux disease; HAPYs, Health Anxiety by Proxy Scale; IIS-P, illness intrusiveness scale parent; IBD, inflammatory bowel disease; IBS, irritable bowel syndrome; MSPSS, Multidimensional Scale of Perceived Social Support; No, number; PedsQL^™^ FIM, Pediatric Quality of Life Inventory^™^ Family Impact Module; PGCIQ, Pediatric GERD Caregiver Impact Questionnaire; PIP, Pediatric Inventory for Parents; POOPC, Parental Opinions of Pediatric Constipation; PREOM-BMP, Patient-Reported Experience and Outcome Measure in a Bowel Management Program; PROM, patient-reported outcome measure; PSS-10, 10-Item Perceived Stress Scale; RAND-36, RAND-36 Item Health Survey 1.0; SB, Spina bifida; SBS, short bowel syndrome; SCL-8, Symptom Checklist-8; SS-P, stigma scale parent; WPAI-CD, Work Productivity and Activity Impairment-Crohn’s Disease; WPAI-UC, Work Productivity and Activity Impairment-Ulcerative Colitis; ZBI-A, Arabic version of the Zarit Burden Interview.

**Table 3:** Characteristics of Included PROMs

PROM Name	Domains/Subscales	Caregiver Population	PROM Language	Domains, No.	Items, No.	Recall Period	Likert or VAS Scale	Score Range	Freely Available
Arabic CDPCA-QOL	Emotional Worries Social	Children with Celiac Disease	Arabic	3	30	Not described	5-point scale	30–150	No
Arabic MSPSS	Social Support	Children with chronic gastrointestinal disorders	Arabic	1	12	Not described	7-point scale	1–7	No
Arabic PSS-10	Perceived Stress	Children with chronic gastrointestinal disorders	Arabic	1	10	Not described	5-point scale	0–40	No
CDLIFE	Social Impact External support Adaptative vigilance Eating adjustments Financial Impact	Children with Celiac Disease	English	5	21	Not described	5-point scale	0–5	Yes
CDPCA-QOL	Emotional Worries Social	Children with Celiac Disease	Portuguese	3	30	Not described	5-point scale	30–150	No
CINCY-FIS	Parental stress	Children with Fecal Incontinence	English	1	4	Not described	5-point scale	5–20	Yes
Croatian PedsQL FIM	Physical, emotional, social functioning Cognitive Communication Worry Daily activities Family relationships	Children with IBD and celiac disease	Croatian	8	36	4-week	5-point scale	0–100	Yes
FAQL-PB	Family, school and social events, time employed to prepare foods, physical and mental state	Children with FPIES	English	17	17	Not described	7-point scale	17–119	No
FIC QOL	Family relationships Emotional impact Financial impact	Children with fecal incontinence, and constipation	English	7	47	Not described	Not described	Not described	No
FS-IS	Daily activities Worry Feeding difficulty challenges	Children with feeding/swallowing problems	English	3	18	1-month	5-point scale	5–90	No
GAD-7	Anxiety	Children of chronic gastrointestinal diseases	English	1	7	14-days	4-point scale	0–21	Yes
HAPYs	Health anxiety by proxy	Youths with functional abdominal pain disorders	English	1	26	2-weeks	5-point	0–104	No
IIS-P	Work Social & family relationships Leisure	Adolescents with IBD	English	3	13	Not described	7-point scale	13–91	Yes
PGCIQ	Physical, emotional, social well-being Caregiving Relationships Daily activities Financial impact Employment	Children with GERD	English Spanish	10	49	Not available	5-point scale	Not available	No
PIP	Medical Care Communication Emotional functioning Role function	Adolescents with IBD	English	4	42	4-week	5-point scale	42–210	Yes
POOPC	Parental burden Family conflict Difficulties with the medical team Worry about Social Impact	Children with constipation	English	4	24	Not described	5-point scale	5–120	No
Portuguese version FS-IS	Family relationships Emotional impact Financial impact	Children with feeding/swallowing problems	Portuguese	3	18	1-month	5-point scale	5–90	No
PREOM-BMP	Physical and emotional concerns Quality of life Financial impact Employment	Children with constipation	English	5	32	Not described	4-point scale	33–132	No
PSS-10	Perceived Stress	Children with chronic gastrointestinal disorders	English	1	10	1-month	5-point scale	0–40	Yes
RAND36	Physical health Emotional Social function Energy/fatigue Bodily pain General health perception	Adolescents with IBD	English	8	36	4-week	5-point scale 6-point scale	0–100	Yes
SBS-modified FaMM (pilot)	Family difficulty Caregiver management ability and effort	Children with SBS	English	4	Not described	Not available	5-point scale	Not described	No
SCL-8	Emotional distress	Youths with functional abdominal pain disorders	English	1	8	1-month	5-point scale	0–32	Yes
SS-P	Stigma	Adolescents with IBD	English	1	8	Not described	5-point scale	8–40	Unknown
WPAI-CD-Caregiver	Productivity Activity Impairment	Children with Crohn’s Disease	Spanish	2	6	7-days	11-point VAS scale	0–100	Yes
WPAI-UC-Caregiver	Productivity Activity Impairment	Children with Ulcerative Colitis	Spanish	2	6	7-days	11-point VAS scale	0–100	Yes
ZBI-A	Personal strain Role strain	Included children with chronic gastrointestinal disorders	Arabic	2	12	Not described	5-point scale	0–48	No

Abbreviations: BMP, bowel management program; CDLIFE, Celiac Disease Life Inventory of Family Experiences; CDPCA-QoL, Celiac Disease Parent Caregiver Quality of Life; CINCY-FIS, Cincinnati Fecal Incontinence Scale; FaMM, Family Management Measure; FAQL-PB, Food Allergy Quality of Life–Parental Burden; FIC QOL, Fecal Incontinence and Constipation Quality of Life; FPIES, food protein induced enterocolitis; FS-IS, Feeding/Swallowing Impact Survey; GAD-7, Generalized Anxiety Disorder-7; GERD, gastroesophageal reflux disease; HAPYs, Health Anxiety by Proxy Scale; IIS-P, illness intrusiveness scale parent; IBD, inflammatory bowel disease; IBS, irritable bowel syndrome; MSPSS, Multidimensional Scale of Perceived Social Support; No, number; PedsQL^™^ FIM, Pediatric Quality of Life Inventory^™^ Family Impact Module; PGCIQ, Pediatric GERD Caregiver Impact Questionnaire; PIP, Pediatric Inventory for Parents; POOPC, Parental Opinions of Pediatric Constipation; PREOM-BMP, Patient-Reported Experience and Outcome Measure in a Bowel Management Program; PROM, patient-reported outcome measure; PSS-10, 10-item Perceived Stress Scale; RAND-36, RAND-36 Item Health Survey 1.0; SB, Spina bifida; SBS, short bowel syndrome; SCL-8, Symptom Checklist-8; SS-P, stigma scale parent; VAS, Visual Analog Scale; WPAI-CD, Work Productivity and Activity Impairment-Crohn’s Disease; WPAI-UC, Work Productivity and Activity Impairment-Ulcerative Colitis; ZBI-A, Arabic version of the Zarit Burden Interview.

**Table 4:** Summary of Findings

PROM	Psychometric measurement property^a^																		Recommendation^c^
	Content Validity		Structural Validity		Internal Consistency		Cross-cultural validity		Reliability		Measurement Error		Criterion Validity		Hypothesis testing		Responsiveness		
	Result	LoE^b^	Result	LoE^b^	Result	LoE^b^	Result	LoE^b^	Result	LoE^b^	Result	LoE^b^	Result	LoE^b^	Result	LoE^b^	Result	LoE^b^	
**Arabic CDPCA-QoL**	+	Mod.	+	Mod.	+	Mod.	+	Mod.	?	NA	?	NA	?	NA	?	NA	?	NA	**Recommend (A)**
**Arabic MSPSS**	?	NA	?	NA	+	Mod	?	NA	?	NA	?	NA	?	NA	?	NA	?	NA	**Research Needed (B)**
**Arabic PSS**	?	NA	?	NA	+	Mod	?	NA	?	NA	?	NA	?	NA	?	NA	?	NA	**Research Needed (B)**
**CDLIFE**	+	Mod.	+	Mod.	+	Mod.	?	NA	?	NA	?	NA	+	Mod	+	Mod.	?	NA	**Recommend (A)**
**CDPCA-QoL**	+	Mod.	+	Mod.	+	Mod.	?	NA	+	Low	+	Low	?	NA	?	NA	+	Low	**Recommend (A)**
**CINCY-FIS**	+	Mod.	+	Mod.	+	Mod.	?	NA	+	Low	+	Mod.	+	Mod	+	Mod.	?	NA	**Recommend (A)**
**Croatian PedsQL FIM**	+	Mod.	+	Mod.	+	Mod.	+	Mod.	?	NA	?	NA	+	Mod.	?	Mod.	?	NA	**Recommend (A)**
**FAQL-PB**	?	NA	+	Low	+	Low	?	NA	?	NA	+	Low	+	Low	+	Low	?	NA	**Research Needed (B)**
**FIC QOL**	+	Low	+	Low	?	NA	?	NA	+	Low	?	NA	?	NA	?	NA	?	NA	**Research Needed (B)**
**FS-IS**	+	Low	+	Mod.	+	Mod.	?	NA	?	NA	?	NA	+	Mod.	+	Mod.	?	NA	**Recommend (A)**
**GAD-7**	?	NA	?	NA	+	Mod	?	NA	?	NA	?	NA	?	NA	?	NA	?	NA	**Research Needed (B)**
**HAPYs**	?	NA	?	NA	+	Mod	?	NA	?	NA	?	NA	?	NA	?	NA	?	NA	**Research Needed (B)**
**IIS-P**	?	NA	?	NA	+	Mod	?	NA	?	NA	?	NA	?	NA	?	NA	?	NA	**Research Needed (B)**
**PGCIQ**	+	Mod.	?	NA	?	NA	?	NA	?	NA	?	NA	?	NA	?	NA	?	NA	**Research Needed (B)**
**PIP**	?	NA	?	NA	+	Mod	?	NA	?	NA	?	NA	?	NA	?	NA	?	NA	**Research Needed (B)**
**POOPC**	+	Mod.	+	Mod.	+	Mod.	?	NA	?	NA	?	NA	+	Mod.	+	Mod.	?	NA	**Recommend (A)**
**Portuguese FS-IS**	+	Mod	+	Mod	+	Mod.	+	Mod.	+	Low	?	NA	+	Mod.	+	Mod.	?	NA	**Recommend (A)**
**PREOM-BMP**	+	Mod.	?	Low	+	Mod.	?	NA	?	NA	?	NA	?	NA	?	NA	?	NA	**Research Needed (B)**
**PSS-10**	?	NA	?	NA	+	Mod	?	NA	?	NA	?	NA	?	NA	?	NA	?	NA	**Research Needed (B)**
**RAND-36**	?	NA	?	NA	+	Low	?	NA	?	NA	?	NA	?	NA	?	NA	?	NA	**Research Needed (B)**
**SBS-FaMM**	+	Low	?	NA	?	NA	?	NA	?	NA	?	NA	?	NA	?	NA	?	NA	**Research Needed (B)**
**SCL-8**	?	NA	?	NA	+	Mod	?	NA	?	NA	?	NA	?	NA	?	NA	?	NA	**Research Needed (B)**
**SS-P**	?	NA	?	NA	+	Mod	?	NA	?	NA	?	NA	?	NA	?	NA	?	NA	**Research Needed (B)**
**WPAI-CD-Caregiver**	+	Mod.	+	Mod.	+	Mod.	+	Mod.	?	NA	?	NA	?	NA	?	NA	?	NA	**Recommend (A)**
**WPAI-UC-Caregiver**	+	Mod.	+	Mod.	+	Mod.	+	Mod.	?	NA	?	NA	?	NA	?	NA	?	NA	**Recommend (A)**
**ZBI-A**	?	NA	?	NA	+	Mod	?	NA	?	NA	?	NA	?	NA	?	NA	?	NA	**Research Needed (B)**

Abbreviations: LoE, level of evidence; Mod, moderate; NA, not assessed; PROM, patient-reported outcome measure.

a Ratings for overall quality for each psychometric measurement property are reported as sufficient (+), insufficient (−), indeterminate (?).

b Assessed using Grading of Recommendations Assessment, Development, and Evaluation, reported as high, moderate, low, or very low.

c Recommendation criteria: A indicates evidence for sufficient content validity (any level; ≥3 of 4 domains) and at least low-quality evidence for sufficient internal consistency (which requires sufficient structural validity); these measures can be recommended for use. B indicates measures not categorized as A or C; these measures require further evaluation to assess quality before being recommended for use. C indicates high-quality evidence for a measurement property rated as insufficient; these measures are not recommended for use.

## Data Availability

All data supporting the findings of this study are available within the manuscript and its Supplementary Information. The literature search strategies are provided in Supplementary Material 2.
